# Prevention, Rehabilitation, and Mitigation Strategies of Cognitive Deficits in Aging with HIV: Implications for Practice and Research

**DOI:** 10.1155/2013/297173

**Published:** 2013-02-03

**Authors:** David E. Vance

**Affiliations:** School of Nursing, University of Alabama at Birmingham, Birmingham, AL 35222, USA

## Abstract

Highly active antiretroviral therapy has given the chance to those living with HIV to keep on living, allowing them the opportunity to age and perhaps age successfully. Yet, there are severe challenges to successful aging with HIV, one of which is cognitive deficits. Nearly half of those with HIV experience cognitive deficits that can interfere with everyday functioning, medical decision making, and quality of life. Given that cognitive deficits develop with more frequency and intensity with increasing age, concerns mount that as people age with HIV, they may experience more severe cognitive deficits. These concerns become especially germane given that by 2015, 50% of those with HIV will be 50 and older, and this older cohort of adults is expected to grow. As such, this paper focuses on the etiologies of such cognitive deficits within the context of cognitive reserve and neuroplasticity. From this, evidence-based and hypothetical prevention (i.e., cognitive prescriptions), rehabilitation (i.e., speed of processing training), and mitigation (i.e., spaced retrieval method) strategies are reviewed. Implications for nursing practice and research are posited.

## 1. Introduction

Highly active antiretroviral therapy (HAART) helps treat and prevent the spread of HIV [1–4] but also provides the opportunity for people to age with this disease [5]. This is welcome news to the 33 million people worldwide who are living with this disease [6]. Still, this population represents an enormous strain on the healthcare system of many countries grappling with this disease. For example, in South Africa approximately 17.8% of those between 18 and 49 are infected with HIV [6, 7], which means they must make difficult decisions in allocating resources and rationing healthcare [8–10]. All of this is occurring within the larger context of worldwide aging. The fastest growing age group on the planet is those 60 and older which constitutes 700 million people, and this cohort will swell to 2 billion by 2050 [11].

**Figure 1:**
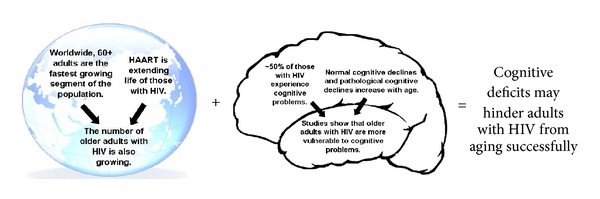
Older adults with HIV will be more at risk for cognitive deficits.

In part due to the unprecedented and historical event of global aging [12] along with HAART increasing the lifespan of those infected with HIV, the number of older adults with HIV is increasing ([5]; Figure 1). In the United States, it is projected that by 2015, half of those with HIV will be 50 and older [13, 14]. Although many are aging with this disease, later-life infections also occur. Those 50 and older account for 15% of all new diagnoses [15]. All of this ushers in new concerns such as how to promote quality of life and facilitate successful aging with HIV.

According to P. B. Baltes and M. M. Baltes [16], successful aging requires competencies in eight areas: length of life, biological health, cognitive efficiency, mental health, social competence, productivity, personal control, and life satisfaction. If one possesses a sufficient amount of these competencies, one is considered to be aging successfully. However, some of these competencies may be more important in promoting successful aging than others. In particular, cognitive efficiency or cognitive health, just like physical health, is often taken for granted until it is compromised. It can be argued that optimal cognitive functioning is needed to help promote biological health (i.e., remembering to take medication [17]), mental health (i.e., being able to exert enough cognitive energy to consciously switch attention from dwelling on negative thoughts to positive thoughts [18]), social competence (i.e., attending to details in conversation [19]), personal control (i.e., decision making such as in finances [20, 21]), and life satisfaction (i.e., remembering and reflecting on personal events and deriving meaning [22–24]). Based on this argument, optimal cognitive functioning is one of the most important competencies needed to help adults with HIV age successfully.

Unfortunately, HIV not only affects the immune system, but it also produces neurological sequelae often resulting in cognitive deficits [25, 26]. In a cross-sectional study, Heaton and colleagues [27] administered a neuropsychological battery to 1,555 adults with HIV (*M*_age_ = 43.2; SD = 8.5) from six sites across the United States. In this sample, 52% of patients exhibited observed cognitive deficits; specifically, 33% exhibited asymptomatic neurocognitive deficits, 12% exhibited mild neurocognitive disorder, 5% exhibited confounded neurocognitive deficits, and 2% exhibited HIV-related dementia. In fact, studies have shown that even after 1 year of being diagnosed with HIV, changes in brain chemistry and metabolism are evident which correspond to poorer cognitive performance [28–30].

Given that increasing age is associated with more cognitive complaints, cognitive deficits, and the risk for pathological cognitive aging (i.e., Alzheimer's disease, vascular dementia, and Lewy body dementia) [31–33], concerns mount that as people age with HIV, they may become more vulnerable for developing such cognitive deficits and dementia (Figure 1; [34–38]). As highlighted in Figure 1, this risk is further increased since HIV produces systemic inflammation and neuroinflammation which may accelerate normal aging and brain aging, respectively [39–42]. In a recent study, Vance et al. [43] administered a comprehensive neuropsychological and everyday functioning battery to four groups of participants: (1) 47 younger adults with HIV (<50), (2) 31 older adults with HIV (50+), (3) 43 younger adults without HIV (<50), and (4) 41 older adults without HIV (50+). These researchers found that as a group, the older adults with HIV performed the worst on all 9 measures; furthermore, significant main effects for both age and HIV were found in a variety of cognitive domains including executive functioning and reasoning, speed of processing, memory, and fine psychomotor ability. 

Similarly, in a sample of 59 adults with and 55 adults without HIV, Baldewicz and colleagues [44] administered a neuropsychological battery every 6 months to them for over 8 years. Over time, those with HIV exhibited significant declines in speed of processing and fine motor speed compared to their HIV-negative counterparts. Also, those who progressed to AIDS (a CD4+ lymphocyte count less than 200 cells/mm^3^) experienced the worst declines in cognitive functioning. Likewise, in a sample of 182 adults with HIV, Valcour and colleagues [45] found that older adults with HIV were nearly three times more likely to develop HIV-associated dementia than younger adults with HIV. This and other studies reinforce the prevalence of such a cognitive vulnerability phenotype in older adults with HIV [17, 36, 46–52].

Thus, the purpose of this paper is to provide an overview on the cognitive sequelae observed in adults with HIV, especially as they age and are more vulnerable to such cognitive deficits. In doing so, the known and possible mechanisms of such cognitive deficits are examined within the context of cognitive reserve. Next, within this context, prevention, rehabilitation, and mitigation strategies are examined; this paper includes both known evidence-based strategies as well as potential strategies that might be effective in augmenting cognition in this population. Finally, general directions and implications for nursing practice and research are posited.

## 2. Cognitive Reserve and Neuroplasticity

**Figure 2:**
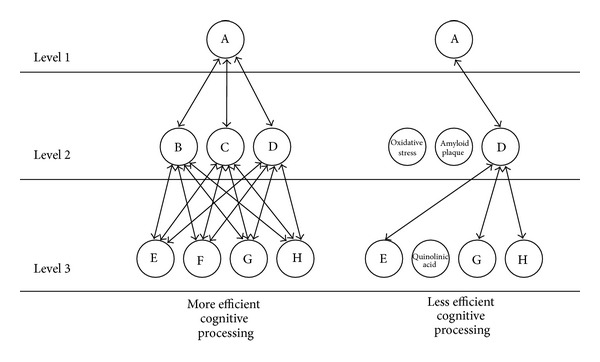
Neural communication and cognitive reserve. Circles represent neurons and arrows represent neural pathways.

The brain's ability to efficiently process information by using existing and alternative neural networks in lieu of damage or disease-related insults (e.g., transient ischemic attack) is referred to as cognitive reserve [53–57]. In its most simplistic form, cognitive reserve refers to the complexity, efficiency, and strength of the neural connections in the brain; it is from these intricate connections in which neurons communicate to each other and convey information that cognition emerges. The more intricate and efficient these connections, the faster neural communication is transmitted and the more complex the thoughts that can emerge from such communication. However, this complexity also produces a protective effect. For example, if some of the neural connections are severed due to disuse, traumatic brain injury, or disease-related insults, information can be rerouted through alternative neural pathways so that neural information can still be communicated and cognition can continue to function. This simplistic explanation can be seen in Figure 2. In the left panel, a neural system that is operating at peak efficiency is represented. Information from neuron A at level 1 is able to transmit neural information through many pathways down to level 3 where it needs to go; however, as indicated by the dark bold lines, there may be a preferred path that is used more consistently which reflects over learning (i.e., routine). This routine path reflects a strong neural pathway. In contrast, in the right panel of Figure 2, the neural system is not operating at peak efficiency. Neurons B, C, and F are compromised by oxidative stress, amyloid plaques, and quinolinic acid, respectively. Because neurons B, C, and F are not functional due to damage or neural death, the number of paths leading from level 1 to level 3 is dramatically reduced. So this neural system will be more vulnerable to cognitive disruption because there are generally less efficient pathways available in communicating neural information. Furthermore, cognitive efficiency would decrease more as the existing neurons are compromised. Thus, the greater one's cognitive reserve (i.e., number and strength of connections between neurons), the more one is able to withstand neurological insults and maintain cognitive efficiency.

Many factors affect cognitive reserve such as health status, stress and negative emotion, substance use, genetics, and environment [53, 58–61]; however, the underlying process that increases or decreases cognitive reserve is positive neuroplasticity and negative neuroplasticity, respectively [62]. Positive neuroplasticity refers to the nervous system's ability to increase the number and strength of connections between neurons in response to novel stimuli. Likewise, negative neuroplasticity refers to the nervous system's ability to decrease the number and strength of connections (i.e., atrophy) between neurons in response to lack of novel stimuli. This process of cognitive reserve and cognition is well documented in animal studies that use the enriched environmental paradigm and human studies involving taxi drivers, older adults, and those with dementia. 

The enriched environmental paradigm used with rats [63, 64] and dogs [65] epitomizes these concepts. For example, genetically similar rats (i.e., from the same colony) are randomly placed to live in one of three conditions: impoverished, standard, or enriched environments. In the impoverish environment, rats are placed one to a cage, so these rats have no other rats with which to socialize and interact. In the standard environment, rats are placed three to a cage, so these rats have other rats with which to socialize and interact. In the enriched environment, several rats are placed to a cage that contains toys for them to explore, so these rats have other rats with which to socialize and interact and have toys with which to explore and interact. Otherwise, the rats receive identical food, water, and other care. After exposure to these environments for a set period of time (i.e., several weeks to several months depending on the study), researchers placed these rats in mazes to test their memory and spatial abilities. Rats placed in the impoverished environment performed statistically worse on maze tasks than those rats placed in the standard environment; likewise, rats placed in the standard environment performed statistically worse on maze tasks than those rats placed in the enriched environment [56, 63]. These findings suggest that being placed in an impoverished environment leads to worse cognitive functioning (i.e., maze performance); this is indicative of negative neuroplasticity. Similarly, these findings suggest that being placed in an enriched environment leads to better cognitive functioning (i.e., maze performance); this is indicative of positive neuroplasticity [61]. Further analyses confirm that changes in brain chemistry and brain morphology correspond to cognitive functioning (i.e., maze performance) in rats. Specifically, more dendritic connections between neurons and higher levels of brain neurotrophic factor were observed in the brains of rats in the enriched environment compared to rats in the standard and impoverished environments; likewise, this same pattern of findings was found in the brains of rats in the standard environment compared to rats in the impoverished environment [66–69]. 

Just as rats were exposed to novel and complex stimuli in the enriched environmental paradigm, humans are also exposed to various levels of novel environmental stimuli that can also produce neuroplastic changes that either increase or decrease cognitive reserve. A seminal study investigating this phenomenon in humans is the London Taxi Driver Study [70, 71]. In this naturalistic study, researchers examined two groups of people (i.e., London taxi drivers and London bus drivers) who underwent two different training conditions for their jobs. In order for London taxi drivers to earn their license, they had to engage in a 2 to 4 year training program whereby they had to familiarize themselves with London's 25,000 streets and learn how to navigate through them, as well as recall many points of interest along the way. This intense training program represents an enriched environment. The London bus drivers did not participate in such an intense training program in order to qualify for their license. In addition, bus drivers were assigned a route that changed little. This lack of training represents a standard or impoverished condition. MRIs of the brains of these taxi drivers were compared to brains of London bus drivers; this revealed significantly larger midposterior hippocampi (brain structures necessary for encoding and consolidating memory) in taxi drivers than bus drivers. Interestingly, the more years of being a taxi driver was associated with larger hippocampi; however, such an association was not found for this group of bus drivers. Thus, this study shows that exposure to novel and challenging stimuli may actually change brain morphology in humans. 

These principles of neuroplasticity and cognitive reserve have also been demonstrated in a seminal study focusing on older adults. Boyke et al. [72] administered a brain MRI (i.e., Time 1) to 69 community-dwelling older adults (*M*_age_ = 60; 50–67) and then attempted to teach them how to juggle in a 3-ball cascade pattern. Approximately 3 months later, after 25 of these participants learned how to successfully juggle for at least 1 minute, brain MRIs (i.e., Time 2) were administered again. Then approximately 3 months later, brain MRIs (i.e., Time 3) of these participants were administered after they stopped juggling. These researchers observed that the nucleus accumbens and hippocampi increased in size from Time 1 to Time 2 as people learned to juggle; this reflects positive neuroplasticity and a change in brain morphology indicative of increased cognitive reserve. Likewise, it was observed that these same brain structures decreased in size from Time 2 to Time 3 as people stopped juggling; this reflects negative neuroplasticity and a change in brain morphology indicative of decreased cognitive reserve. 

These studies demonstrate that exposure to stimulating and enriching activities promotes positive neuroplasticity and may increase cognitive reserve. Meanwhile, these studies also provide evidence that being exposed to nonstimulating and nonenriching activities promotes negative neuroplasticity and may decrease cognitive reserve. In fact, many studies are investigating how these principles of neuroplasticity and cognitive reserve may be used to protect against age-related cognitive impairment and dementia [24, 54, 73, 74]. For example, Roe and colleagues [75] investigated whether educational attainment, an indicator of being exposed to stimulating activities that may increase cognitive reserve, is related to the delay in the onset of cognitive symptoms of Alzheimer's-related dementia in a sample of 37 adults with and 161 adults without this disease. Using radiotracers and positron emission tomography to measure the amount of fibrillar beta-amyloid (i.e., amyloid plaques) as an indicator of disease severity, these researchers observed that even in the presence of high levels of fibrillar beta-amyloid which impedes neural communication (Figure 1), those with high levels of education (and essentially more cognitive reserve) performed better cognitively. Thus, this study suggests that even when amyloid plaques may reduce the number of connections between neurons, remaining neural connections, which reflect cognitive reserve, allow information to be rerouted to other neurons which supports continued cognitive functioning. This rerouting process of cognitive reserve is observed in other systems in the body. For example, in occlusive coronary heart disease where blood vessels are blocked, coronary collateral arteries circumvent such blockage by providing an alternative route for circulation, which reduces the risk of myocardial ischemia [76]. By having such alternative and redundant function, such bodily systems, whether they are nervous or circulatory, can continue in lieu of diseases and insults. Such information becomes quite germane considering the variety of insults that can compromise cognitive reserve in those aging with HIV [77].

## 3. Mechanisms of Cognitive Deficits

**Figure 3:**
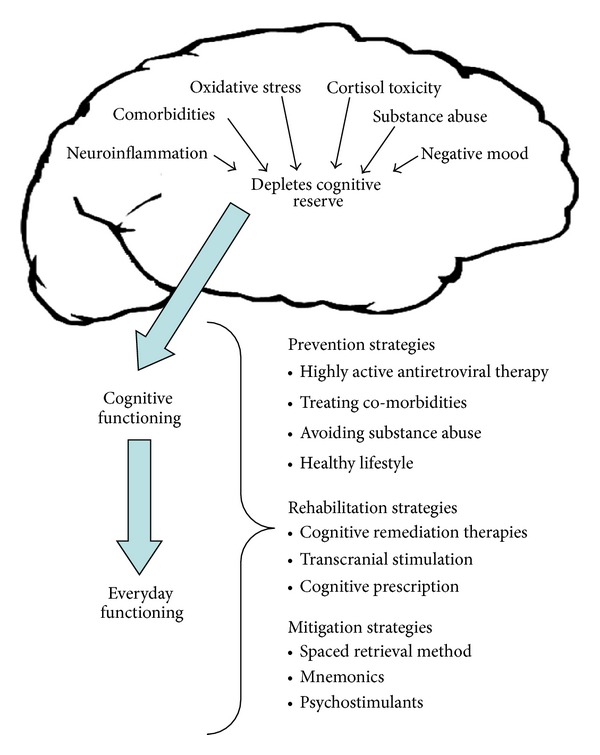
The effects of HIV on brain function and preventive, rehabilitative, and mitigation strategies.

As highlighted in Figure 3, several mechanisms endemic to HIV and normal aging can be damaging to the brain, deplete cognitive reserve, and diminish cognitive functioning. Although by no means exhaustive, some of the most widely recognized mechanisms are neuroinflammation; comorbidities; oxidative stress; and cortisol toxicity, negative affect, and substance abuse.

### 3.1. Neuroinflammation

Inflammation is an immune response to pathogens, disease, injury, and oxidative stress and is a fundamental component in the healing process. Although normally a good biological process, prolonged inflammation causes tissue damage and can result in neurodegeneration and impair neurogenesis [78]. Unfortunately, even when HIV is controlled through HAART, the body still responds to the virus by producing cytokines (i.e., molecules that increase or decrease immune response) that modulate the immune system, thus creating inflammation in the body and neuroinflammation in the brain. In fact, the accelerated aging observed in HIV is often attributed to this inflammation; for this reason, HIV is considered a “slow burner” inflammation disease as well as an immunodeficiency disease [79]. 

The link between neuroinflammation and cognitive functioning has been well established [78, 80–82].

For example, in a large sample of older adults, Yaffe and colleagues [83] examined the association between cognitive functioning as measured by the Modified Mini-Mental State Examination and inflammation as measured by serum levels of inflammatory molecules (i.e., IL-6, TNF-*α*, and CRP) at baseline and over 2 years. These researchers found that those with the highest tertile of CRP and IL-6 at baseline and 2 years later performed significantly poorer on this cognitive test at baseline and 2 years compared to those in the lowest tertile for these inflammatory markers. In fact, those in the highest tertile had a 24% increased risk of cognitive decline over 2 years. 

Similarly, this neuroinflammation observed in HIV has been attributed to many of the cognitive deficits that are observed in this population [84]. To exemplify this, Thompson and colleagues [85] compared MRI brain scans of 26 adults with HIV treated with HAART to 15 adults without HIV. These researchers found that even in these medically stable adults with HIV, thinning was observed in the prefrontal parietal cortices compared to the control group. Such cortical thinning is attributed to the inflammation and neuroinflammation that is occurring with this disease. Thus, addressing neuroinflammation may be an area ripe for cognitive intervention. 

### 3.2. Comorbidities

That which affects the body also affects the brain. Given this basic principle, it is not surprising that comorbidities such as heart disease, liver disease such as hepatitis C, and renal disease also affect neurological and cognitive functioning [86]. For example, untreated hypertension leads to small-vessel cerebrovascular disease that creates white matter lesions; these small lesions are basically damaged neural tissue [87, 88]. Using MRIs, de Leeuw and colleagues [87] found that in a sample of 1,077 community-dwelling older adults (*M*_age_ = 51; 60–90 years) the duration of hypertension corresponded to more periventricular and subcortical white matter lesions. In addition, these researchers found that those adults with untreated hypertension experienced more of such lesions in both of these brain areas.

In another example, Bruehl and colleagues [89] compared the cognitive functioning of 41 adults with type 2 diabetes to 47 adults without type 2 diabetes matched on age, gender, and education. These researchers found that those with type 2 diabetes performed more poorly on cognitive tests compared to those without type 2 diabetes. In fact, MRI scans revealed that those with type 2 diabetes also had decreased volume in their prefrontal cortex (the brain region needed for executive functioning and reasoning) and hippocampi (the brain structures needed for memory). Further analysis revealed that the decreased volume of the prefrontal cortex was statistically correlated to poorer glycemic control. This study and several others [90, 91] revealed a similar association between comorbidities and cognitive functioning [86]. Such comorbidity and cognitive deficits are of concern given that HAART can create or exacerbate such comorbidities and that such comorbidities (e.g., heart disease and hypercholesterolemia) are highly prevalent in older adults with HIV [49, 92–94].

### 3.3. Oxidative Stress

Oxygen is needed by the body to perform a host of functions, yet oxygen itself is a corrosive element and damages tissues. In particular, reactive oxygen species, sometimes referred to as free radicals, interact with tissues and produce a variety of cellular and molecular changes that cause inflammation [95]. To counteract the detrimental effects of such reactive oxygen species, the body utilizes an intricate system of antioxidant mechanisms whereby such effects are minimized or rendered inert. Sadly, such antioxidant mechanisms become less efficient with increased age. As such, these reactive oxygen species damage tissues; this is frequently referred to as oxidative stress [96]. In particular, reactive oxygen species can damage mitochondria (i.e., the energy producing organelle in cells) which subsequently impair cell function. But reactive oxygen species can also progressively damage DNA over time. Since DNA is used to generate cells and tissue, this accumulated damage results in less efficient tissues being produced resulting in poor systemic functioning. In fact, the effects of oxidative stress on mitochondria and DNA are one of the prevailing theories on the causes of aging [52, 95]. 

The brain, being composed of neural tissue, is also negatively affected by oxidative stress. This effect has been observed in well-controlled rat and human studies. Singh and colleagues [97] compared rats that received food enriched with folic acid, an antioxidant, over an 8-week period to same age rats that received standard food. These researchers found that those rats that received folic acid performed better than their counterparts on maze, active avoidance, and passive avoidance tests which served as indicators of cognitive functioning. Upon further examination, lipid perioxidant, a proxy of aging, was significantly lower in the brains of the folic acid fed rats. This and other studies suggest that foods rich in antioxidants can be neuroprotective and support cognitive reserve [98, 99].

In humans, this protective relationship has also been observed. In a sample of 1,389 older adults (60–70 years), Akbaraly and colleagues [100] examined plasma selenium (an antioxidant) levels and cognitive functioning over 9 years. Using a series of linear and logistic models, these researchers found that a decline in selenium was associated with a decline in cognitive functioning. With the abundance of evidence supporting antioxidant supplementation on brain health and cognition, such supplementation could provide some protection against age-related neurodegenerative diseases [101–104].

### 3.4. Cortisol Toxicity, Negative Affect, and Substance Abuse

HIV is a stigmatizing disease that can produce an intense emotional reaction for those infected [105]. Unfortunately, one's emotional state, specifically experiencing severe negative affect or mood, can obviously impact cognition in at least three ways: (1) it increases cortisol levels, (2) it competes for existing cognitive resources, and (3) it can result in poor coping techniques (i.e., substance abuse). First, a negative emotional state is associated with increased cytokines (an inflammatory molecule) and stress hormones like cortisol that over time can cause inflammation which can impair or damage neural tissue [106]. In particular, stress, anxiety, and depression can lead to a response of the hypothalamic-pituitary-adrenal axis whereby glucocorticoids (i.e., cortisol) are secreted [107]. Although cortisol is needed by the body to suppress the immune system, increase energy production, and help metabolize proteins, fats, and carbohydrates during periods of stress [108], prolonged exposure can damage the brain, in particular hippocampal neurons [109, 110]. In a cross-sectional study of 967 older adults (50–70 years), Lee and colleagues [111] found that higher levels of salivary cortisol levels were predictive of poorer cognitive functioning across multiple domains (i.e., speed of processing, verbal memory, visual memory, learning, and executive functioning). In a longitudinal study of 538 older adults (70–79 years), Karlamangla and colleagues [112] found that higher levels of urinary cortisol levels at baseline were predictive of poorer cognitive functioning seven years later. Furthermore, this association was still observed after taking into consideration age, gender, education, blood pressure, smoking, and cardiovascular disease. Thus, such dysregulation of the hypothalamic-pituitary-adrenal axis and subsequent long-term exposure of cortisol may slowly deplete cognitive reserve and increase the risk of developing cognitive deficits. 

Second, ruminating over such negative affect can compete for cognitive resources that otherwise could normally be allocated to other tasks [18]. For example, it is difficult to concentrate on and switch attention to a complex accounting problem (e.g., balancing one's checkbook) when one is also dwelling on a severe emotional problem that is consuming one's mental resources. In fact, many studies clearly demonstrate that depression, stress, and anxiety are associated with poorer cognitive functioning [86]. Using the analogy of a computer virus, negative affect can slow down speed of processing which can detrimentally impact cognitive functioning. Sadly, as a group, older adults with HIV do experience considerable negative affect and generally have a fragile social network to help cope with such aversive emotional states [113]. For example, in a sample of 914 older adults with HIV, Grov and colleagues [114] observed that 63% of their sample experienced clinically significant levels of depressive symptomatology. Similarly, Kalichman et al. [115] found in a sample of 113 middle-aged and older men with HIV that 27% thought about taking their life within the past week; clearly, such suicidal ideation can consume normal rational thought processes. Ostensibly, negative affect is present in this clinical population and it may be severe enough to interfere with cognitive functioning.

Third, poor coping skills with such negative affect can also lead to poorer cognitive function such as through engagement with alcohol and substance use [116–118]. Current and past alcohol and substance abuse can produce obvious neurological problems that negatively impact cognitive functioning [119, 120]. In particular, glial cells, which support neuronal functioning and aid in forming connections between neurons, are compromised by many substances; this leaves neurons more vulnerable to oxidative stress and cortisol toxicity [53, 121]. Alcohol and substance abuse has also been shown to reduce hippocampi size and cognitive functioning in adolescence; because of this reduced growth and diminished cognitive reserve, such adolescents may be more vulnerable to developing cognitive deficits in later life [122]. In a sample of 98 adults with HIV, Fazeli and colleagues [123] found that self-reported alcohol and substance use was predictive of poorer cognitive functioning. This and other studies support these general findings [47, 77].

## 4. Prevention Strategies

Implementing strategies of protecting cognition is not often considered until one observes such loss in a friend or family member. Yet, prevention strategies to avoid cognitive loss are obviously preferred rather than relying on rehabilitation and mitigation strategies that may be only moderately effective. Although by no means exhaustive, a few of the more salient strategies of preventing cognitive deficits in adults aging with HIV are receiving HAART, treating comorbidities, treating depression and anxiety, avoiding substance abuse, and engaging in a healthy lifestyle [38].

### 4.1. HAART

Treating HIV with HAART so that the virus does not compromise the immune system and thus prevent opportunistic infections, which can also impact brain health, is the most obvious strategy for preventing cognitive deficits in this population [124]. In 59 adults with HIV who were either HAART-naïve or about to change their HAART regimen due to treatment failure (i.e., the medication was no longer suppressing viral replication), Parsons et al. [125] assessed the cognitive functioning of these participants at baseline (before taking HAART) and six months later (after taking HAART). These researchers found that those participants who achieved successful viral suppression also experienced improvements in their cognitive functioning compared to those who did not achieve successful viral suppression.

Despite HAART's ability to protect and improve cognitive functioning in adults with HIV [126, 127], four caveats must be considered when using these medications. First, the HAART medications are not equally effective in crossing the blood brain barrier; in other words, some medications are more efficient at entering the brain and hindering the detrimental effects of HIV than others [128]. For example, within one of the class of HAART medications known as nucleoside reverse transcriptase inhibitors, the medications tenofovir, zalcitabine, and didanosine do not cross the blood brain barrier well while zidovudine and abacavir do so very well. Therefore, prescribing HAART medications to address cognitive deficits must take this penetration of the blood brain barrier into consideration. (For more information, Letendre and colleagues [128] provide a schema for which HAART medications have lower, intermediate, and high blood brain barrier penetration.)

Second, a high adherence rate (i.e., 95%) to HAART is needed to achieve viral suppression and avoid drug resistance and viral mutation [129, 130]. Unfortunately, poor medication adherence can lead to cognitive problems [17]; likewise, cognitive problems can lead to poor medication adherence [131–133]. For these reasons, effective strategies are needed to remember to take one's medications consistently. (For an example of such medication adherence strategies, see the spaced retrieval method in the Mitigation Strategies section of this paper.)

Third, some of the HAART medications can be neurotoxic. For example, efavirenz, a nonnucleoside reverse transcriptase inhibitor, increases the risk of developing cognitive deficits [134]. Although the exact mechanism of these neurotoxic effects is unknown [126], it has been suggested that nonnucleoside reverse transcriptase inhibitors in particular may adversely affect brain metabolism and damage neuronal mitochondria [30, 126, 135].

Finally, HAART creates metabolic syndromes that can either cause or exacerbate comorbidities such as hypercholesterolemia, hypertension, heart disease, and diabetes [94]. These comorbidities have been shown to negatively impact brain health and cognitive functioning [86]. In addition, such comorbidities can produce extra stress on other bodily systems by increasing inflammation and oxidative stress which also impairs neurons [78]. 

### 4.2. Treating Comorbidities

As mentioned, the presence of comorbidities exacts a burden on the brain resulting in less cognitive reserve and more cognitive deficits. As such, preventing such comorbidities through healthier lifestyles and treating such comorbidities through medication to restore physiological homeostasis represent a practical strategy for maintaining or improving cognitive functioning. Treating comorbidities with the appropriate medication has been shown in several studies to exert cognitive benefits [86]. For example, in a sample of 18,999 older women (70–81 years), Logroscino et al. [136] examined the effects of different type 2 diabetes treatments on cognitive functioning over a two-year period. These researchers found that those women with type 2 diabetes treated with oral hypoglycaemic agents performed at the same level cognitively to women without type 2 diabetes at baseline and two years later; however, women with type 2 diabetes treated with insulin performed poorer cognitively in comparison. Meanwhile, those women with type 2 diabetes not being treated cognitively performed the worst in general at baseline and two years later.

Similarly, although it is well established that hypertension and heart disease can negatively impact cognitive functioning [137–139], treating such conditions medically can mitigate their affect. Viamonte and colleagues [140] examined the cognitive functioning of 315 community-dwelling older adults without heart disease to 185, 41 and 56 community-dwelling older adults treated for hypertension, heart disease, or a combination of both, respectively. These researchers found that there were no cognitive differences between these groups; this and other studies [141, 142] support the idea that medically treating such comorbidities as hypertension can yield cognitive benefits or protect against the development of cognitive deficits. Thus, with the average number of comorbidities for adults with HIV in their 50s and +60s being 4.40 and 4.52, respectively [94], addressing such comorbidities is an obvious way to support cognitive reserve and cognitive functioning. 

### 4.3. Treating Depression and Anxiety

The prevalence of depression and anxiety across each decade of life for adults with HIV hovers around 40% and 20%, respectively [94]. As already mentioned (see Cortisol, Negative Affect, and Substance Abuse section), there is a strong (and possible causal) connection between negative mood and cognition in this clinical population [143]. In fact, in a sample of 107 adults with HIV, Thames and colleagues [144] found that those who experienced elevated levels of depression reported more cognitive problems. Thus, treating psychiatric comorbidities may also be important for protecting and improving cognitive functioning [145]; in fact, several studies show this. Claypoole and colleagues [146] found that in a sample of 78 adults with HIV suffering from depression and treated with antidepressants for 12 weeks reported significantly fewer cognitive problems and improved on objective measures of cognitive functioning. Given the strong connection between negative mood and cognitive functioning via the hypothalamic-pituitary-adrenal axis [107], treating psychiatric comorbidities such as depression and anxiety should be considered as a potential treatment of cognitive deficits in this clinical population.

### 4.4. Avoiding Substance Abuse

Substance abuse has been shown in several studies to negatively impact cognitive functioning in normal adults and adults with HIV [123, 147, 148]. Yet, some studies do not support these findings; surprisingly, these findings are mixed [149, 150]. For example, in a sample of 399 adults with HIV categorized into three groups of substance users (134 with no substance use, 131 with nonsyndromic substance use, and 134 with syndromic substance use matched on CD4 nadir, depressive symptomatology, and literacy level), Byrd and colleagues [151] found out the same prevalence of cognitive deficits across these groups, regardless of substance use engagement. In fact, those with a history of cocaine or marijuana use actually had higher levels of verbal fluency. In making sense of these findings, it is important to note that substance use is a complex behavior. For example, some adults who abuse substances may have more cognitive reserve and can withstand such neurological insults than others; thus, cognitive deficits may not always manifest in lieu of substance abuse, and this could explain some of the mixed findings. Another likely explanation of these mixed findings is that many adults who once engaged in substance abuse may have experienced cognitive recovery once abstinent or reduced the level of substance use. For instance, Iudicello and colleagues [152] followed 83 methamphetamine users over a 1-year period and found that those who were able to abstain performed similarly to a healthy matched control while those who continued using did not show cognitive improvement. Other studies also confirm cognitive recovery after abstinence or reduction of use for alcohol, marijuana, and other substances [153–155]. Finally, it has been shown that the recency of substance abuse may be the strongest predictor of experiencing cognitive deficits [156, 157]. Given this evidence, it becomes more certain that not only avoidance of substance abuse is recommended, but reducing or abstaining can improve cognitive functioning.

### 4.5. Healthy Lifestyle

Related to avoidance of substance abuse, a healthy lifestyle can produce positive outcomes to one's quality of life, to the body, and to the brain. The impact of physical exercise, mental exercise, healthy social interactions and social support, good nutrition, and good sleep on brain health and cognition cannot be emphasized enough [49, 158–160]. In fact, some of these healthy lifestyle factors can also reduce inflammation and oxidative stress that can be damaging to the brain [49, 53]. For example, in several studies, foods rich in polyphenols and antioxidants, which can reduce such oxidative stress and inflammation, have been found to be associated with better cognitive functioning in older adults [161–163]. In fact, green tea, which is rich in polyphenols, has been found to reduce astrogliosis in HIV-1 tat-transgenic mice [164]. Thus, similar cognitively beneficial effects for diet as well as other healthy lifestyle factors may also be observed in adults with HIV. 

## 5. Rehabilitation Strategies

Cognitive deficits that may occur when aging with HIV may be mild or moderate enough such that rehabilitation strategies may be effective in restoring cognitive functioning to near previous levels. But it is also important to note that cognitive deficits may emerge because of transitory conditions (e.g., poor sleep hygiene, negative mood, and thiamine deficiency) that may be addressed by just some simple changes in lifestyle [38]; for that reason, cognitive prescriptions are listed as a potential rehabilitation strategy. Although by no means exhaustive, two other rehabilitation strategies presented (i.e., cognitive remediation therapy and transcranial stimulation) represent novel ways of bolstering positive neuroplasticity and cognitive reserve. 

### 5.1. Cognitive Prescriptions

Given the advantages that a healthy lifestyle exerts on cognitive health, Vance and colleagues [165] have incorporated such advantages into a holistic, individualized behavioral program, called cognitive prescriptions, that may be used to support cognitive reserve and cognitive functioning. In this regard, it may be considered as both a preventative and rehabilitation strategy. Cognitive prescriptions target several lifestyle areas known to affect cognitive functioning; these include intellectual exercise, physical exercise, social engagement, mood support, sleep hygiene, good nutrition, and substance use. In its most basic form, the nurse clinician uses motivational interviewing techniques [166] and dialogues with the patient to determine what goals he or she wishes to pursue in each lifestyle area; from this, specific behavioral goals are developed with clear objective steps for reaching such goals utilizing standard behavioral therapy techniques [167]. Such goals are represented in a highly visual way and displayed in a prominent place (i.e., refrigerator and bathroom mirror) to encourage compliance and facilitate tracking of outcomes. In addition, if certain goals are achieved or need to be altered, the program can easily accommodate the change of old goals for new goals. 

For example, if the patient enjoyed painting years ago but has gotten out of the habit but is still interested in doing so, the goal may be to paint one hour a week or paint one 5 × 5 inch painting a week (whatever the patient thinks is appropriate); it is important to make the goals very specific and concrete. This is a goal that may serve in either the intellectual exercise area or, if it is relaxing to the patient, the mood support area. In another example, the patient may be experiencing bouts of insomnia which can obviously impact cognitive functioning [168]. Thus, a goal may be to (1) go for a 30-minute walk every evening and (2) take a warm bath before going to bed to relax. Again, motivational interviewing techniques are used to determine that these are intrinsically appealing activities for the patient. As before, these goals may serve simultaneously in the physical exercise area, the mood support area, and the sleep hygiene area. The advantages of cognitive prescriptions are that they represent a concrete way of supporting positive neuroplasticity and cognitive health [24, 55, 56] while simultaneously addressing other areas to promote overall health and quality of life.

### 5.2. Cognitive Remediation Therapy

Cognitive remediation therapy represents a collection of various strategies with the expressed purpose to either improve a particular cognitive ability such as memory, executive functioning and reasoning, and psychomotor ability or improve overall cognition. It consists of specially designed cognitive exercises that can also be delivered through several venues such as pencil-and-paper, videotape/DVDs, computer programs (i.e., gaming technology), and class-type workshops [169, 170]. For example, in a sample of 159 community-dwelling normal older adults (65–92), Vance and colleagues [171] randomized these participants into two groups—the internet training (control) condition and the speed of processing training (experimental) condition. Those in the internet training condition engaged in approximately ten hours of exercises designed to improve their skills in learning how to use the worldwide web; however, these exercises were only designed to increase factual knowledge of the internet and not to improve any fluid cognitive abilities. Those in the speed of processing training condition engaged in approximately ten hours of computer exercises designed to improve their visual speed of processing and visual attention as measured by a computer test called the Useful Field of View (UFOV) test. These speed of processing exercises administered in a game-type format required these participants to identify quickly presented objects (i.e., between 17 and 500 milliseconds) in the center of the screen and locate a peripheral object on the screen. Using a double-staircase method in the program, when participants answered incorrectly, the presentation speed was automatically reduced for the next trial, but when participants answered correctly, the presentation speed was automatically increased for the next trial. Thus, this training program forced participants to use their visual speed of processing abilities at their maximum level, and thereby pushed their threshold ability to improve. It was found that those who engaged in the speed of processing training condition improved on measures of visual speed of processing and attention (i.e., UFOV and Starry Night) compared to those in the internet training condition. In addition, the treatment effects were observed even two years after training ceased.

This study is particularly germane to adults with HIV. Marcotte and colleagues [172] found that in a sample of 68 adults with HIV that half experienced cognitive deficits in visual speed of processing as measured by UFOV. This finding is of particular concern given that deficits observed on UFOV tend to increase with age and are found to be associated with poor driving outcomes [170, 173]. Thus, compromised driving skills, as well as other everyday functions that also depend on this cognitive ability, may be a concern as adults continue to age with HIV. 

Fortunately, cognitive remediation therapy has been used, similar to the previous study protocol [171], to improve speed of processing in middle-aged and older adults with HIV (*M*_age_ = 51.55; range 40.7–70.6). In this pre-post two-group experimental design study, Vance et al. [174] randomized these 46 adults into either a speed of processing training condition (*n* = 22) or to a no-contact control condition (*n* = 24). Like the previous study, those in the speed of processing training condition received approximately 10 hours of training over a 4-to-6-week period. Compared to the control condition, those assigned to the speed of processing condition significantly improved on UFOV as well as the Timed Instrumental Activities of Daily Living test, a measure of speeded everyday functioning such as looking up phone numbers and identifying items on a shelf of food. Although more work must be done in determining if these findings are consistent with a larger HIV-positive sample and whether the training will improve functioning in other areas such as medication adherence or driving behavior, cognitive remediation therapy represents a viable and noninvasive approach for rehabilitating cognitive abilities in those experiencing cognitive deficits.

However, a caveat about using such cognitive remediation therapies is warranted. Not all cognitive remediation training programs commercially available are the same. Some are designed to improve general cognition while others target particular cognitive abilities such as memory. In selecting such a cognitive remediation therapy, one must be mindful of the research and evidence supporting their use and effectiveness given that some have been tested more thoroughly than others [170, 175].

### 5.3. Transcranial Stimulation

Transcranial stimulation is a strategy in which either magnetic currents or electrical currents are applied to the scalp over certain brain regions with the goal of enhancing cognitive functioning or in some cases inhibiting certain maladaptive behaviors such as addictions or depression [176–178]. In this paper, focusing more on transcranial direct current stimulation (tDCS), because of relatively cheaper cost and easier access and use than transcranial magnetic stimulation, administration of this particular strategy has been shown to improve cognitive functioning in several studies [179–183]. For example, Lindenberg and colleagues [184] examined brain activity patterns during a motor activity in 20 stroke patients with hemiparesis (i.e., weakness on one side of the body) before and after occupational/physical therapy with half randomized to receive five concurrent sessions with tDCS and the other half randomized to receive five concurrent sham sessions (i.e., without tDCS). Occupational/physical therapy, in conjunction with tDCS, was found to increase neural activity of the ipsilesional motor cortex and resulted in improved motor function as assessed by the Upper Extremity Fugl-Meyer instrument.

Unfortunately, the exact mechanism in which tDCS increases (or in some cases decreases) such neural activity is not fully understood. It has been suggested that tDCS is able to connect remote regions of the brain which may possibly boost existing cognitive ability [179]. More specifically, Nitsche [185] hypothesized that such direct current alters the electrical current across neural membranes. Anodal stimulation (versus cathodal stimulation) in particular flows from the electrode into the scalp and into the brain, pulling the electrical currents of neurons across the surface of the cerebral cortex a few millivolts making them more prone to depolarization. Thus, when signals arrive from other neurons, they are more responsive and more likely to fire which can aid in learning and perhaps promote positive neuroplasticity. In support of this hypothesis, increased activity in the cerebral cortex has been found after utilizing tDCS. 

In a recent study, Clark and colleagues [181] used a series of single-blind, randomized studies to examine the effects of tDCS on learning a complex visual task (i.e., identifying concealed objects) in 96 healthy adults. Participants played a virtual reality environment game called “DARWARD Ambush!” in which they had to identify where lethal objects such as bombs were concealed in the pictures based on feedback. These researchers fastened a 10 cm^2^ sponge electrode over the right inferior frontal cortex near F10 (i.e., directly over the sphenoid bone). Participants received the direct current 5 minutes before starting the exercise and 30 minutes within the training. Clark and colleagues found that anodal 2.0 milliamp current resulted in significantly better learning that 0.1 milliamp exposure. Other studies have also found that anodal 2.0 milliamp exposure over F10 and F8 (i.e., left prefrontal cortex) may be effective in improving learning and that the effects on learning may last for several hours afterwards [179, 180].

Although tDCS has not been used to boost cognitive functioning in adults with HIV, it has been considered as a possible treatment for depression [186]. In a small sample of 8 adults with HIV suffering from depression, Knotkova and colleagues [187] administered tDCS anodal current over the F3 area (the region of the scalp that corresponds to the dorsolateral prefrontal cortex) and the cathodal current over the contralateral supraorbital region at 2.0 milliamps for 20 minutes for 8 to 10 sessions over consecutive days. These researchers found that depression scores significantly decreased; in fact, they also found that the Mini-Mental State Exam scores improved or remained unchanged. Perhaps because depression can compete for cognitive resources, abating depression may indirectly boost cognitive functioning [86]. Although this is a small pilot study, it does show that tDCS was well tolerated. Given that tDCS was shown to improve learning and cognition in normal adults as seen in Clark and colleagues' [181] study, perhaps this approach could be used with older adults with HIV to rehabilitate cognitive functions that may have declined. 

## 6. Mitigation Strategies

Once cognitive deficits develop, if rehabilitation strategies are not effective in improving existing cognitive abilities, then mitigation strategies should be considered to compensate for the loss of cognitive functioning. Fortunately, the gerontological literature is replete with several cognitive tools such as spaced retrieval method and numerous mnemonics which can easily be translated to those aging with HIV. In addition, some psychopharmacological agents such as psychostimulants may be used to bolster cognitive functioning for brief periods; unfortunately, the use of such medication-related mitigation strategies is not without some potential side effects and other patient costs. 

### 6.1. Spaced Retrieval Method

**Figure 4:**
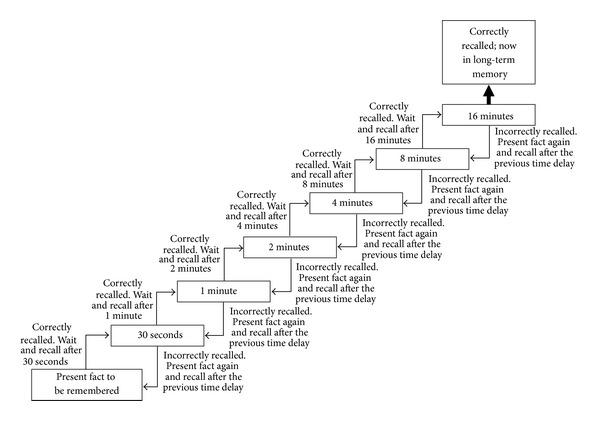
Stair step approach of the spaced retrieval method.

The spaced retrieval method is a formalized memory strategy used to help people retain a discrete unit of information or fact such as a telephone number or an address [188]. The key to this strategy is to recall the information over progressively longer periods of time until it is finally consolidated into one's long-term memory (Figure 4). For example, the fact to be remembered is presented and then repeated back immediately, then after 30 seconds, 1 minute, 2 minutes, 4 minutes, 8 minutes, and finally 16 minutes. In this stair step recall procedure, if the fact cannot be recalled at any time, then the fact to be remembered is presented again to the person and then it is to be recalled after the previous time point in which the person successfully recalled the fact. This back and forth process continues until the information can be successfully recalled after 16 minutes; once the fact can be recalled after 16 minutes without cues, the information is fully consolidated into one's long-term memory. 

Spaced retrieval method has been used in many clinical populations with memory problems including those with aphasia, traumatic brain injury, and early stage dementia [189, 190]. Neundorfer and colleagues [191] used a combination of spaced retrieval method and external mnemonics (i.e., notes and lists) to help 10 older adults with HIV (*M*_age_ = 50; 50–60) who had executive functioning and memory problems to perform two everyday tasks (e.g., remembering to take medications on time and remembering to attend clinic appointments). These researchers delivered this intervention in eight 30-minute sessions over 4 weeks. In general, the combination of spaced retrieval method and external memory aids was found to be effective in helping those participants compensate for their cognitive problems and successfully complete their everyday tasks. Since this strategy is simple to use and does not require any special equipment, it represents a viable tool for those older adults with HIV experiencing cognitive deficits that interfere with everyday functioning. 

### 6.2. Mnemonics

As just mentioned, spaced retrieval method is a very specific mnemonic that has been shown to be helpful in compensating for cognitive deficits. However, several other mnemonic strategies may also be helpful for older adults with HIV including method of loci, chunking, and external memory aids. All of these strategies are easy to use and do not require any special equipment. 

**Figure 5:**
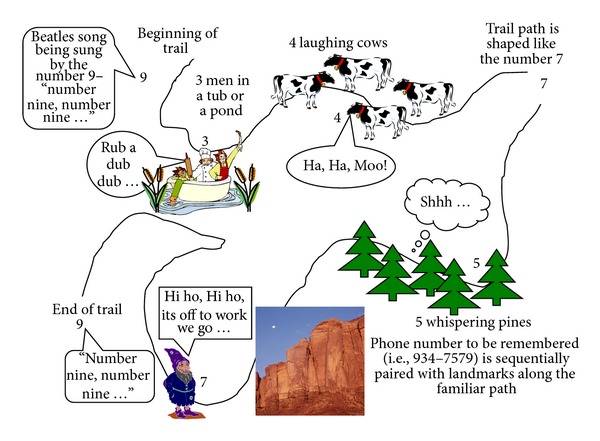
Example of how to use method of loci.

Method of loci is a mnemonic for remembering sequential information by visualizing the information to be remembered with landmarks of a familiar path or items within one's home or a regular routine [192]. For example, as seen in Figure 5, if one needs to memorize a phone number, one may visualize the numbers along a familiar trail. So if the number is 934–7579, one can imagine that a large nine is singing the Beattle's song “Number 9” at the trail head; then along the path one can imagine 3 men in a tub in the pond nearby. Further down the path, one can imagine 4 loud laughing cows in a meadow and then image how the trail seems to curve as to make the number 7. As one continues down the path, one can imagine 5 whispering pines and then a 7 at a large outcropping of rocks and then another number 9 concluding with singing the “Number 9” song. The more novel, vivid, and detailed one makes these associations, the more likely the associations will be successfully recalled. For example, with the outcropping of rocks, one can imagine seven dwarves living in them. Likewise, method of loci could be used to assist with HIV disease management such as pairing watching the nightly news with taking one's HIV medication; that way when someone watches the evening news as part of their normal routine schedule, they can use the news program as a mnemonic to remember to take their medication on time.

Chunking is another technique one can employ to recall information [192, 193]. Using a phone number as another example, to memorize the same series of number (i.e., 934–7579), one can memorize the first three digits as a chunk and then the last four digits as a chunk and then assemble them together. In fact, this is how many actors memorize long monologues, by committing separate sections to memory in chunks and then weaving them together. Similarly, in memorizing one's medication regimens, one may memorize one medication protocol at a time and then put them together.

Finally, external memory aids can be quite effective and easy to use strategies to recall information [192]. It can be something as simple as a checklist on one's refrigerator detailing as to when to take one's medications, or it can be an app on a cell phone reminding one to attend his or her medical appointment, or it can even be a partner/friend encouraging one to check his or her blood pressure. Much of this is self-explanatory and falls within the realm of “common sense”; however, its usefulness and utility in assisting with everyday functioning for those suffering from cognitive deficits cannot be underscored enough.

### 6.3. Psychostimulants

Two psychostimulants have been found to improve cognitive functioning in adults with HIV: methylphenidate (Ritalin) and modafinil. Although methylphenidate has not been studied in adults with HIV in a large randomized clinical trial, several studies have found it to improve cognition in normal healthy adults [194], those with multiple sclerosis [195], and those with attention deficit disorder [196]. In a placebo-controlled, single-blind crossover study involving 16 adults with HIV, Hinkin and colleagues [197] found that methylphenidate (once daily 30 mg dose) abated cognitive slowing as measured by a computerized neuropsychological measure. Methylphenidate may improve cognitive functioning in adults with HIV in one of two ways. First, methylphenidate is a dopamine agonist [196]. Given that some adults with HIV experience lower levels of dopamine [198], methylphenidate may be an effective strategy to increase levels of dopamine in the brain and thus mitigate such cognitive deficits. Second, methylphenidate has also been shown to lower the levels of triglycerides, low density lipoprotein cholesterol, and total cholesterol [199]. Since higher cholesterol levels are associated with more cognitive deficits in adults with HIV [200] and 65.8% of older adults (60+) with HIV experience higher levels of hypercholesterolemia [94], methylphenidate represents another strategy for addressing such cognitive problems by treating this common comorbidity.

Modafinil has also been shown to improve cognitive functioning and abate fatigue in adults such as in those with attention deficit disorder [201]. In a heterogenous sample of 103 adults with HIV (51%, 40%, and 20% with a substance use history, depression diagnosis, and a hepatitis C diagnosis, resp.), McElhiney et al. [202] randomized participants to receive 4 weeks of modafinil or a placebo. These researchers found that those assigned to the modafinil condition experienced more objective global cognitive gains and fewer subjective cognitive complaints compared to those in the placebo condition. In addition, no adverse effects were observed; thus, this supports the use of modafinil as a strategy to mitigate such cognitive deficits.

Although psychostimulants such as methylphenidate and modafinil have been shown to improve cognition in adults including those with HIV, other medications such as acetylcholinesterase inhibitors, herbal and alternative supplements, omega-3 fatty acids, and hormone replacement therapy may not be as effective in mitigating such cognitive deficits. Acetylcholinesterase inhibitors are traditionally used to treat adults with Alzheimer's disease and related dementias. However, in a sample of 168 community-dwelling older adults without dementia but with memory complaints, Yesavage and colleagues [203] administered donepezil (an acetylcholinesterase inhibitor) over one year and found that it was not effective in improving cognitive functioning. This study suggests that this medication may only be effective in those with dementia, not those with cognitive deficits. Likewise, although some studies suggest that herbal and alternative supplements such as ginseng and ginkgo biloba may protect against cognitive deficits or even improve cognitive functioning, in general such strategies have not been found to be effective [204–206]. In fact, such herbal and alternative supplement may interfere with HAART, medication adherence, and medical treatment [130, 207]. Omega-3 fatty acids have also been considered as a possible avenue for enhancing cognitive functioning given that it is has been shown to be important for brain development and synaptic plasticity. Unfortunately, the findings have been mixed; clearly more research is needed in this area [208]. 

Finally, similar to herbal and alternative supplements, hormone replacement therapy has been touted by some as a possible strategy to prevent dementia and improve cognitive [209, 210]; unfortunately, in general both estrogen and testosterone replacement therapies have been shown to be ineffective in improving cognition in adults [211, 212]. In fact, some studies suggest that estrogen replacement therapy in women may actually increase the risk for dementia by 2-fold [213] and that testosterone replacement therapy in men may increase the risk for liver disease, heart failure, prostate cancer, hyperviscosity, and erythrocytosis [214].

## 7. Implications for Nursing Practice

As mentioned, 52% of those with HIV experience some degree of cognitive deficit [27]; however, this means that 48% do not and function within normal parameters. Clearly, some individuals with HIV are more resistant to such cognitive deficits. Therefore, one of the most important points of nursing practice is to monitor cognitive functioning in adults with HIV to determine who is experiencing such problems [215]. Such monitoring can occur in a number of ways. 

First, nurses can administer cognitive screeners or cognitive self-report instruments to patients such as the Mini-Mental Status Exam [216], the Montreal Cognitive Assessment [217], or the Cognitive Failures Questionnaire [218]. These are simple-to-use tools and require little training in administering and interpreting results. Second, nurses can assess for cognitive changes by observing patients' behaviors such as forgetting medical appointments, word finding difficulty, and outward signs of confusions (e.g., walking in the wrong direction to leave the clinic). If patients have a caregiver, friend, or partner that accompanies the visit, questions regarding cognitive status can be directed to them about their loved ones' cognitive functioning (e.g., Have you seen any change in your friend's memory over the past three months? Does your partner get lost easily?). Finally, patients can be asked directly to report any perceived problems with memory, attention, and cognitive functioning. If patients report such problems, referrals to a psychologist or neurologist can be made. A caveat about this last approach is that patients may not be accurate about their self-appraisal of their cognitive functioning. In fact, many adults with HIV also experience metacognitive problems; in other words, they have difficulty thinking about and evaluating their own thinking [219, 220]. For example, in a sample of 46 adults with HIV, Hinkin and colleagues [221] administered a neuropsychological battery to this sample along with asking them to rate their cognitive ability. From this, nearly 1/3 (37%) overestimated their cognitive ability and nearly 1/3 (26%) underestimated their cognitive ability; meanwhile, nearly 1/3 (37%) accurately estimated their cognitive ability which matched their actual objective cognitive functioning. As such nurses must be aware that even when assessing patients' cognitive functioning, caution should be used in relying solely on self-report since it may not be accurate; thus, a combination of assessments strategies would be preferred to triangulate and validate cognitive functioning and determine the need for specific prevention, remediation, or mitigation strategies. (For a decision tree as to how to assess cognitive functioning in patients with HIV, see Vance et al. [220] for more details.)

Another important point of nursing practice in this area is to convey to patients that the health and lifestyle decisions that they make today will influence the amount of cognitive reserve they will have to weather neurological insults in later life. For those aging with HIV who are more susceptible to heart disease, hypercholesterolemia, diabetes, and other conditions known to impact cognitive functioning [86], this point cannot be emphasized enough. Thus, preventive strategies as discussed in this paper should be suggested as a way of not only protecting cognitive functioning, but may be a way to also ameliorate physical health and improve quality of life. 

## 8. Implications for Nursing Research

Although many areas of research have already been suggested as potential research vectors to prevent, rehabilitate, and mitigate cognition in adults with HIV, several areas of research must continue to examine such strategies to help older adults with HIV age successfully; this is very important given that successful cognitive aging is needed to effectively engage in disease management (e.g., medication adherence and attending clinic visits) and everyday functioning (e.g., safe driving behavior). Lindl et al. [48] highlighted several interesting strategies that may be considered to address cognition in future research. These include nanoparticle delivery of HAART, insulin and insulin-like substances, lithium, and memantine. 

As mentioned earlier, not all HAART medications are effective in crossing the blood brain barrier and delivering the medication to where it needs to be [128, 222]; this is unfortunate since the brain is considered an HIV reservoir [223]. To confront this problem, researchers are considering nanoparticle delivery of HAART so that it can cross the blood brain barrier better resulting in more bioavailability of the medication to control HIV [48, 224, 225]. If effective, this approach could potentially reduce the impact HIV has directly on the brain, not to mention that it could reduce the number of HIV reservoirs in the body. 

Insulin, insulin-like growth factor-1, and neurotrophin intranasal delivery has been suggested as a way to compensate for the neural damage caused by diabetes, stroke, diabetes, Alzheimer's disease, and HIV. Small preliminary studies have been encouraging in that both short-term memory and long-term memory have been improved along with mood and odor detection [48, 226]. This is especially relevant because as adults age with HIV, they may also experience olfactory and related chemosensory declines as well [227, 228]. In a recent double-blind study of 104 adults with mild cognitive impairment or mild-to-moderate Alzheimer's disease, Craft and colleagues [229] randomized participants to receive 4 months of intranasal delivery of (1) 20 international units of insulin a day, (2) 40 international units of insulin a day, or (3) a placebo. In this intent-to-treat analysis, these researchers found those who received the 20 international units of insulin showed improved memory and those in either insulin condition had better preserved cognitive functioning compared to those in the placebo condition. Although this has not been tested in adults with HIV, this does represent a promising vector of research.

Lithium has been suggested as a possible medication to improve cognitive functioning in adults with HIV. In a small 12-week pilot study with 8 adults with HIV experiencing cognitive deficits, Letendre and colleagues [230] found that an open-label use of lithium (300 mg daily) resulted in improved cognition. Similarly, in a small 10-week pilot study with 15 adults with HIV also with cognitive deficits, Schifitto and colleague [231] did not find cognitive improvement with an open label use of lithium (300 mg twice daily); however, these researchers did find several changes in biomarkers (i.e., increased fractional anisotropy) reflective of improved brain health. Although lithium use may not be appropriate for everyone (i.e., those who are pregnant or with renal disease [196, 232, 233]), these initial findings are encouraging because the medication was well tolerated in both studies.

Memantine has also been suggested as a possible medication to improve cognitive functioning in adults with HIV since memantine decreases glutamate excitotoxicity in both Alzheimer's disease and HIV-disease models in mice. In a pilot study with 140 adults with HIV, Schifitto and colleagues [234] found that after 16 weeks of taking memantine (10 mg daily escalated to 40 mg daily), magnetic resonance spectroscopy showed that memantine may exert protective properties for frontal white matter and the parietal cortex. Unfortunately, cognitive benefits were not observed during this 16-week period; however, additional time on memantine may be needed before benefits are observed. In a similar study involving a sample of 99 adults with HIV with cognitive deficits, Zhao and colleagues [235] randomized the participants to receive either memantine (escalated up to 40 mg daily) or a placebo. These researchers found that those in the memantine condition experienced cognitive improvement after 12 weeks compared to those in the placebo condition. Out of these 99 participants, 45 were followed over a 48-week period; unfortunately, the cognitive improvements were no longer detected but this could have been due to developed tolerance to the medication which suggests that it may be useful in only the short term. Fortunately, memantine was well tolerated in both studies and represents an area in need of more investigation.

Studies must also consider how to improve cognitive functioning to improve everyday functioning. As mentioned, Marcotte and colleague [172] observed that in comparison with those without HIV, adults with HIV experience more severe deficits in Useful Field of View—a cognitive measure of visual attention and visual speed of processing. These cognitive abilities are obviously necessary for safe driving and needed to avoid motor vehicle crashes. There is growing concern that as adults with HIV age, these cognitive abilities will atrophy and predispose them for unsafe driving [236]. Fortunately, Ball et al. [237] found that in a sample of 908 community-dwelling older adults without HIV those who received speed of processing training had a 50% reduction in at-fault motor-vehicle crashes six years after training compared to those randomized to the control group. Since this speed of processing training was shown to improve Useful Field of View in a pilot study sample (*n* = 22) of middle-aged and older adults with HIV (see Cognitive Remediation Therapy section [174]), such cognitive remediation therapy may improve driving safety in this cognitively vulnerable group; however, more research is needed on a larger sample of drivers to determine the effectiveness of such therapy on driving. 

## 9. Conclusion

Although the process of aging along with HIV can have negative consequences on brain health through oxidative stress, neuroinflammation, and other mechanisms, this does not mean that all individuals with this disease will experience cognitive deficits. In fact, although Heaton and colleagues [27] found that 52% of adults with HIV experienced some degree of cognitive deficits, it is important to note that 48%, nearly half, do not. Clearly, there are many individuals aging with this disease who are less vulnerable to the detrimental effects aging and HIV exert on the brain. So there is room for optimism concerning successful cognitive aging with HIV; in fact, studies investigating why certain individuals age well cognitively with this disease are obviously warranted. Perhaps such fortunate individuals have greater cognitive reserve or have engaged in preventative strategies (i.e., healthy lifestyle) to avoid cognitive deficits. For those who unfortunately experience such cognitive deficits, hope is not lost either. Several rehabilitative strategies such as speed of processing training and mitigation strategies such as spaced retrieval method have been shown to be effective in this clinical population. Likewise, other strategies such as transcranial direct current stimulation has been shown to benefit older adults [238] and such approaches may be of use to those aging with HIV. Regardless, more prevention, rehabilitation, and mitigation cognitive research is needed to evaluate the effectiveness of such strategies as the number of older adults with HIV grows.
